# An artificial intelligence-based model for prediction of clonal hematopoiesis variants in cell-free DNA samples

**DOI:** 10.1038/s41698-025-00921-w

**Published:** 2025-05-20

**Authors:** Gustavo Arango-Argoty, Marzieh Haghighi, Gerald J. Sun, Elizabeth Y. Choe, Aleksandra Markovets, J. Carl Barrett, Zhongwu Lai, Etai Jacob

**Affiliations:** Oncology R&D, AstraZeneca, Waltham, MA USA

**Keywords:** Biomarkers, Cancer, Computational biology and bioinformatics

## Abstract

Circulating tumor DNA is a critical biomarker in cancer diagnostics, but its accurate interpretation requires careful consideration of clonal hematopoiesis (CH), which can contribute to variants in cell-free DNA and potentially obscure true tumor-derived signals. Accurate detection of somatic variants of CH origin in plasma samples remains challenging in the absence of matched white blood cells sequencing. Here we present an open-source machine learning framework (MetaCH) which classifies variants in cfDNA from plasma-only samples as CH or tumor origin, surpassing state-of-the-art classification rates.

## Main text

Blood-based liquid biopsies are increasingly being explored for personalized cancer care, as circulating tumor DNA (ctDNA) can be detected in circulating cell-free DNA (cfDNA) of blood plasma liquid biopsies^1^. The noninvasive, potentially longitudinal detection of tumor somatic variants from ctDNA could enable early diagnosis, improved biomarker detection and treatment selection, and disease monitoring^2–4^.

However, accurate interpretation of ctDNA variants remains challenging, largely due to the presence of clonal hematopoiesis (CH) in cfDNA. CH refers to somatic variants in hematopoietic cells acquired over a lifetime, often affecting genes associated with aging, as well as those commonly altered in hematological malignancies and solid tumors, such as TP53^5^. While high allele frequency variants can often be filtered out as likely germline or somatic non-tumor, recent evidence suggests that many low allele frequency variants arise from CH and not tumors^6^. CH variants comprise of over 75% of cfDNA variants in individuals without cancer, and sometimes more than 50% of cfDNA variants in those with cancer^6^. Using cfDNA to diagnose, treat, or monitor cancer depends on accurately distinguishing CH variants from true tumor-derived mutations^7^.

Studies characterizing the presence of variants in blood plasma cfDNA commonly utilize matched sequencing of a nucleated blood cell fraction (e.g. peripheral blood mononuclear cells or white blood cells) to determine variant origin (tumor or CH) in the absence of a tumor biopsy^6,8–10^. However, sequencing matched WBCs at the scale and depth required for clinical applications is often cost-prohibitive, time consuming, and impractical to implement^11,12^. Sequencing coverage must match cfDNA assay sensitivity, and WBCs require extra care due to their fragility compared to red blood cells. Matched WBC sequencing also has limitations—CH is a dynamic process, where certain clones might exist in peripheral blood at levels below the detection threshold of standard sequencing yet still contribute detectable mutations to cfDNA. CH variants can be missed due to low variant allele frequency (VAF) in the WBC fraction, clonal heterogeneity, or sampling bias. Additionally, matched WBCs may not be available for retrospective studies or archived plasma samples. When only blood plasma sequencing is available, conventional analyses identify variants as CH by using reference databases of known variants associated with CH or hematological malignancies^10,13^, but many CH variants are specific to individuals or otherwise non-recurrent^6,8^, limiting the sensitivity of this approach. The VAF of key hematologic cancer driver genes has also been suggested as way to identify CH variants in cfDNA samples, but the exact relationship between VAF and variant origin remains unclear^14,15^. These challenges have driven interest in using machine learning (ML)-based methods to classify cfDNA variants^11,16,17^. While nascent, they show promise for further development of ML-based models for variant classification.

**Fig. 1:**
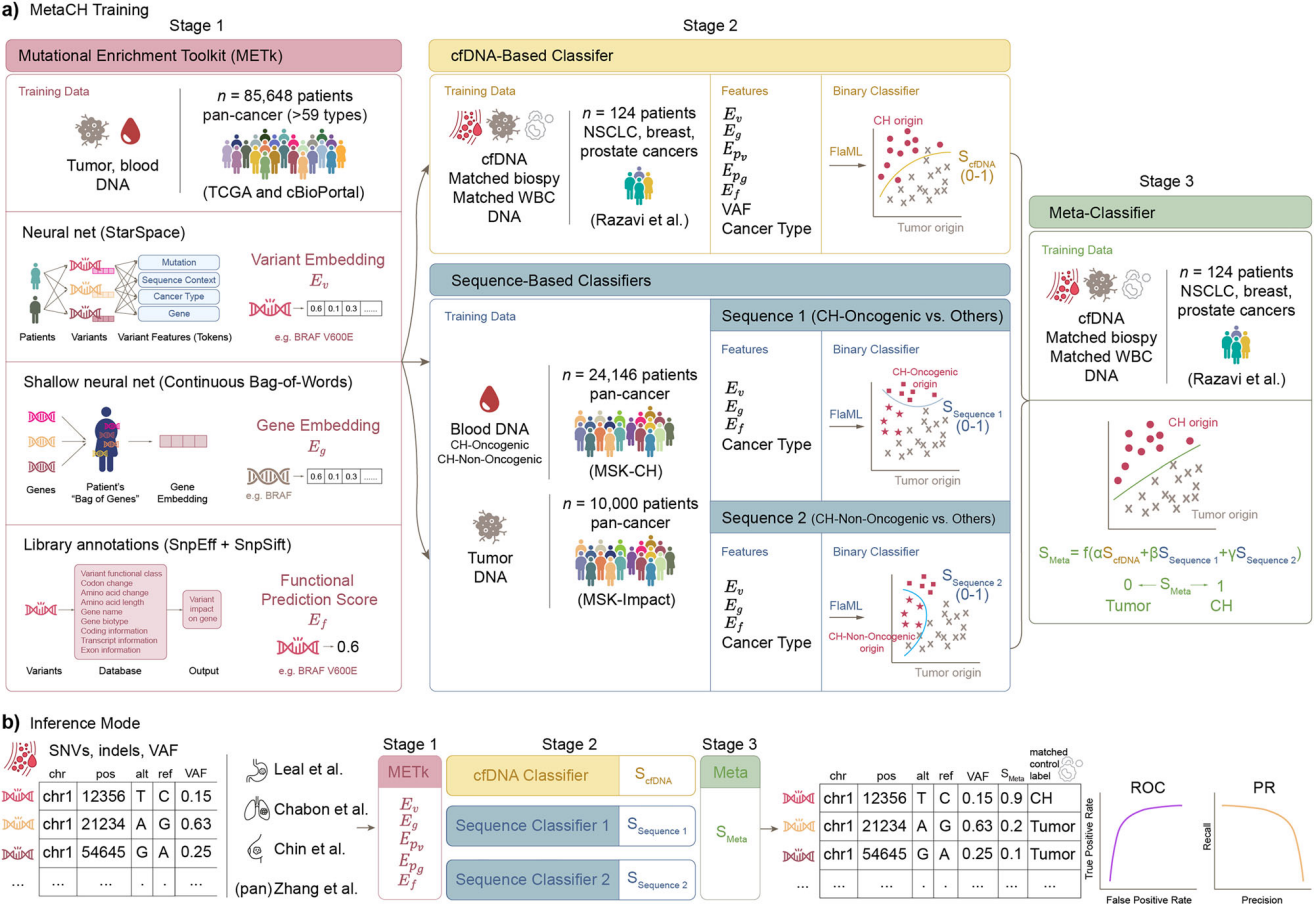
The MetaCH framework. **a** Training the three stages of MetaCH: Stage 1: Mutational Enrichment Toolkit (METk) is trained on tumor and blood sequencing from a large, pan-cancer dataset to learn to generate variant (${E}_{v}$) and gene (${E}_{g}$) embeddings and functional prediction scores (${E}_{f}$). Stage 2: Base classifiers include a cfDNA-based classifier trained on a small cfDNA dataset with experimentally derived annotations (Razavi et al.) and two sequence-based classifiers trained on large datasets of pan-cancer tumor sequencing and blood sequencing with CH subtype annotations. The Sequence 1 classifier distinguishes CH-Oncogenic variants from others (tumor or CH-Non Oncogenic) and Sequence 2 distinguishes CH-Non Oncogenic variants from others (tumor or CH-Oncogenic). Stage 3: A meta classifier trained on Razavi et al. integrates base classifiers scores into a single variant origin prediction score (0: tumor; 1: CH). **b** Inference and validation: SNVs and indels from a cfDNA sample are processed through feature extraction and classification stages, yielding a single score predicting variant origin. Performance is evaluated using auROC and auPR curves on cfDNA datasets representing various cancer types, with ground truth derived from matched WBC sequencing.

Here, we propose MetaCH (a Metaclassifer for Clonal Hematopoiesis detection), an ML-based framework that accurately classifies CH variants in cfDNA samples in the absence of matched sequencing of WBCs. MetaCH processes variants through three stages and returns their corresponding CH-likelihood scores (Fig. 1a).

In the first stage, variants, genes, and the impact of a variant on gene function are numerically represented via the Mutational Enrichment Toolkit (METk) (Fig. 1a). METk extracts three categories of features (Fig. 1a, see Methods for details): variant embeddings (${E}_{v}$), gene embeddings (${E}_{g}$), and functional prediction scores (${E}_{f}$). Variant embeddings (${E}_{v}$) are learned through a self-supervised entity representation model inspired by StarSpace^18^, which maps variants into a shared embedding space based on their sequence context, associated gene, and cancer type. Gene embeddings (${E}_{g}$) capture patterns of genes with variants within individual patients, where each patient is represented as a bag of their genes with variants. This approach, inspired by word embeddings in natural language processing (NLP), learns numerical representations by leveraging co-occurrences of genes with variants within the same patient. The averaged embeddings of all genes with variants for a patient is denoted as ${E}_{{p}_{g}}$, and the averaged embeddings of all variants for a patient is denoted as ${E}_{{p}_{v}}$, providing a compact representation of the patient’s mutation profile. Functional prediction scores (${E}_{f}$) quantify the impact of non-synonymous variants on gene function using publicly available databases and annotation tools (SnpEff, SnpSift) that integrate multiple prediction algorithms. These extracted features—variant and gene embeddings, and functional prediction score—provide information on the variant signature landscape, gene prevalence in cancer, and the functional effect of the variants to classifiers in the second stage.

In the second stage, three base classifiers are trained using METk-generated features. First is a cfDNA-based classifier that learns to distinguish the origin of variants in the context of the cfDNA patient’s samples (“cfDNA-Based Classifier”, Fig. 1a) and returns a CH-likelihood score (S_cfDNA_). It is trained on a smaller, publicly-available dataset from Razavi et al.^6^, in which variants are annotated using cfDNA and paired tumor- and WBC-matched sequencing. This cfDNA-based classifier employs several features: gene (${E}_{g}$), variant (${E}_{v}$), and patient-level embeddings (${E}_{{Pg}}$, which represents the averaged embeddings of all genes within a patient and ${E}_{{Pv}}$, which represents the averaged embeddings of all variants within a patient), and functional variant scores (${E}_{f}$) from METk, as well as variant allele frequencies (*VAF*) and cancer type (*Ct*) for each patient.

The second and third binary base classifiers are sequence-based classifiers which take advantage of large public datasets of tumor and blood-derived genomic sequencing to score the CH-likelihood of variants (“Sequence-Based Classifiers”, Fig. 1a). Unlike cfDNA datasets with matched WBC sequencing, which are smaller than typical cancer genomic datasets and potentially limit model generalizability across cancer types and patient populations, we trained these additional second and third classifiers using two publicly available datasets for CH (blood-derived)^19^ and somatic tumor (cancer-derived)^20^ variants from the Memorial Sloan Kettering Cancer Center. These datasets together comprise 77,068 tumor-derived and 9810 blood-derived variants spanning 59 cancer types. Following prior work^19^, we removed duplicate variants and annotated CH variants into two subgroups based on their putative role in cancer pathogenesis or recurrence in myeloid neoplasms. This resulted in a final three-class dataset of putative cancer driver (CH-Oncogenic) variants, variants not-related to cancer pathogenesis (CH-Non-Oncogenic), and tumor variants. The Sequence 1 classifier predicts CH-Oncogenic from other variants (tumor or CH-Non-Oncogenic, S_Sequence 1_) and Sequence 2 predicts CH-Non-Oncogenic from others (tumor or CH-Oncogenic, S_Sequence 2_).

Finally, the last stage (“Meta-Classifier”, Fig. 1a) is a meta-classifier trained by applying each of the base classifiers to the cfDNA dataset from Razavi et al.^6^ to generate scores (S_cfDNA_, S_Sequence 1_, S_Sequence 2_) representing the probability of each variant having blood (CH) origin. It uses logistic regression to optimally combine the scores from the base classifiers as meta-features into one, final S_Meta_ score. This score represents the probability that a variant originates from CH (1) or tumor (0).

In the inference mode (Fig. 1b), given a pool of cfDNA variants, the three stages of the MetaCH framework process the data and predicts a single CH likelihood score for each variant.

**Fig. 2:**
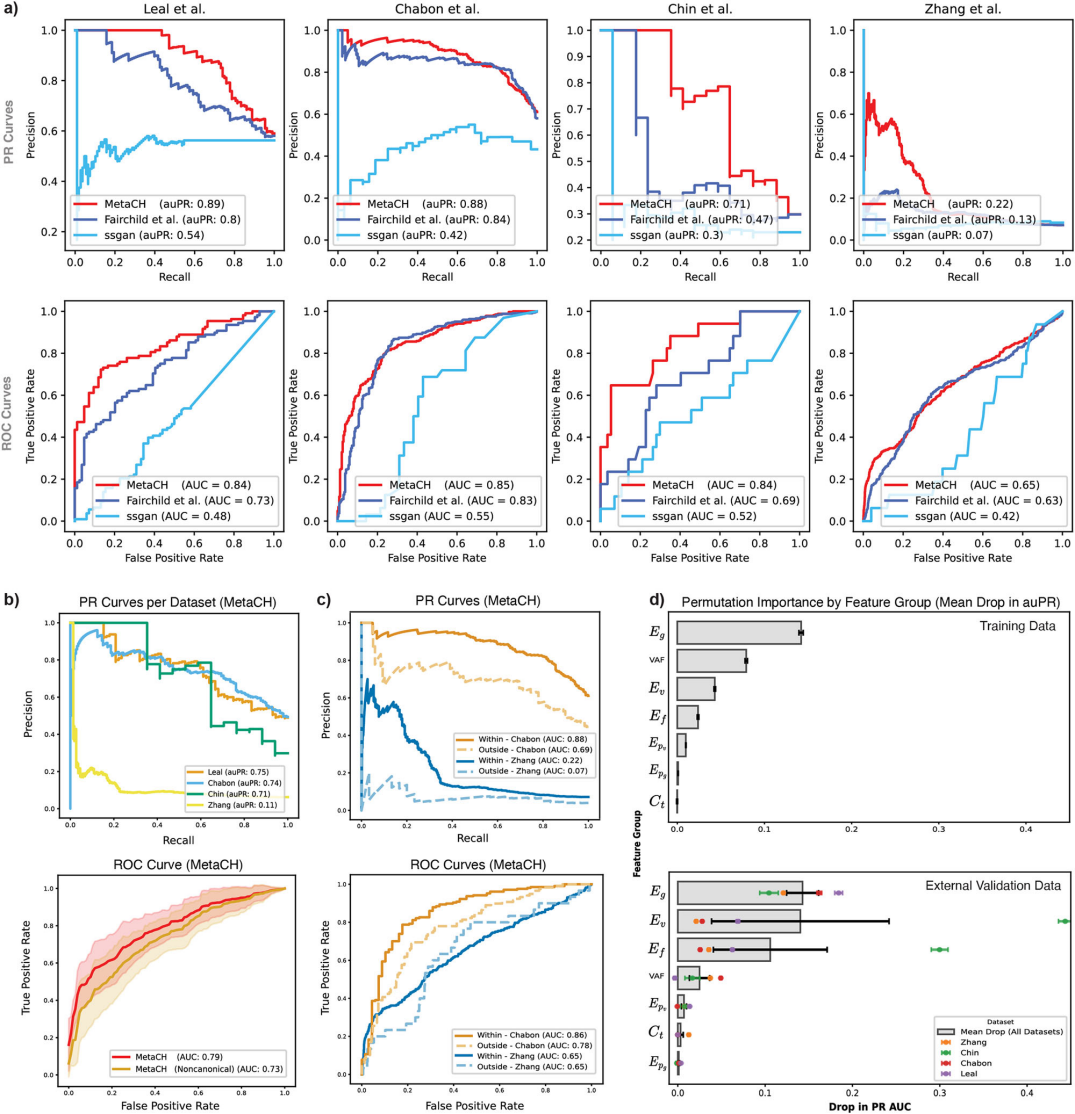
Performance evaluation of MetaCH on external validation datasets. **a** Performance comparison of our proposed model (MetaCH) versus the machine-learning based baseline models for CH classification reveals that the proposed model consistently outperforms the baseline models in identifying the origin of variants across four independent validation datasets according to the area under the precision-recall and ROC curves shown in the first and second rows respectively (denoted by auPR and auROC). **b** Per dataset PR curves (first row) with average ROC curves (second row) comparing MetaCH’s performance with and without canonical CH genes across external validation datasets. MetaCH achieves an auROC of 0.79 when canonical CH genes (DNMT3A, TET2, and ASXL1) are included and an auROC of 0.73 when these genes are excluded from the external validation datasets. ROC curves illustrate overall model performance on the external validation datasets, with shaded areas representing the standard deviation across datasets. **c** PR (first row) and ROC (second row) curves comparing MetaCH’s performance on subsets of the Chabon et al.^8^ and Zhang et al.^21^ datasets containing genes within and outside the Razavi et al.^6^ training panel. **d** Permutation feature importance by feature group, measured as mean drop in auPR. Feature importance is assessed across training and external validation datasets, with each bar representing the mean drop in auPR when permuting a specific feature group. In the training data, gene embeddings ($E_g$) and VAF emerge as the most influential features. In external validation data, variant embeddings ($E_v$) show the highest importance, though with substantial variability across datasets. Gene embeddings ($E_g$) exhibit lower variability, indicating their consistent importance across both training and validation datasets. Error bars represent standard deviation across permutation repetitions (*n* = 30) for the training dataset and standard deviation across datasets for the external validation plot.

We evaluated the performance of the full framework and the standalone performance of each base classifier using cross-validation of the Razavi et al.^6^ training samples and reported the results as both area under the Precision-Recall (auPR) and the Receiver Operating Characteristic (auROC) curves in Supplementary Table 1. The cfDNA-based classifier achieved comparable auPR and auROC as the complete MetaCH framework, despite being trained on the smallest dataset. This underscores the importance of training on cfDNA data with ground truth from matched sequencing data. The complete MetaCH framework showed noticeable advantages when applied to four independent external cfDNA validation datasets with matched WBC sequencing (Supplementary Fig. 1)^8,21–23^. In the Chabon et al. dataset, the cfDNA-based classifier exhibited the highest auPR of the subclassifiers, while in Leal et al., Chin et al., and Zhang et al., it was a sequence-based classifier. However, the MetaCH framework consistently delivered the highest auPR (or one comparable to the highest) across these four datasets compared to the subclassifiers. MetaCH also outperformed existing machine learning approaches^11,16^. Across all external validation datasets, MetaCH demonstrated superior performance in predicting variant origin (Fig. 2a, Supplementary Table 2).

Interestingly, the classifier designed to differentiate CH-Oncogenic variants from others exhibited higher auROC and auPR compared to the CH-Non-Oncogenic classifier (Supplementary Table 1). This suggests that CH-Oncogenic variants are easier to distinguish from tumor variants than CH-Non-Oncogenic variants. This observation could be explained by the distinct genetic signatures associated with different variant types. CH-Oncogenic variants, while precancerous, exhibit variant fingerprints strongly correlated with myeloid lineage and aging, as noted in Bolton et al.^19^, whose classification we adopted for our annotations. In contrast, tumor variant fingerprints are influenced by a wider array of factors, including environmental exposures (e.g. tobacco use, UV radiation) and tumor-specific mutational processes (e.g. APOBEC activity)^24^, leading to greater variability in their variant signatures. CH-Non-Oncogenic variants, being minimally myeloid-derived, may be associated with a broader spectrum of variant signatures, including ones overlapping with tumor, making them harder to classify^25^. This pattern suggests that our model’s ability to capture differences in variant landscapes, in both the sequence-based classifiers and embeddings learned in stage 1, can help indicate the origin of the variants^8,24,26^.

To assess our model’s dependence on the most prevalent CH-associated genes and its ability to generalize to less common CH variants, we evaluated its performance on an external validation set where all variants in the most prevalent genes in clonal hematopoietic cells (*DNMT3A*, *TET2*, and *ASXL1*)^27^ were removed. MetaCH’s performance dropped by approximately 6% (Fig. 2b), indicating that while these genes contribute to classification, they do not disproportionately influence outcomes, and the model still retains predictive capability based on other features.

To assess potential bias towards variants in genes within the training set panel, we evaluated model performance on subsets of the Chabon et al. and Zhang et al. containing genes within and outside the training panel. Results reveal a bias towards training set panel genes (Fig. 2c) and highlight the need for more panel-diverse annotated cfDNA samples in future training sets. Despite this bias, the model maintained predictive power beyond random classification, even for out-of-panel genes.

Finally, we evaluated the importance of each feature category in the MetaCH model using a permutation importance test (Fig. 2d). While VAF emerged as the most influential feature in the training data, its importance varied across validation datasets and was, on average, less than that of METk-extracted features. This highlights the contribution and generalizability of these embeddings, which learn from a large, complementary data source and add important information beyond what can be learned from matched cfDNA sequencing alone. Overall, gene embeddings (${E}_{g}$) were the most informative category for determining variant origin across both training and external validation datasets. Variant embeddings (${E}_{v}$) and functional prediction scores (${E}_{f}$) showed considerable variability in importance across various external validation datasets.

MetaCH performance varied across the datasets, likely due to differences in variant-calling and processing methods, sequencing depth and coverage, sample preparation and sequencing protocols, as well as patient populations. As a result, the model may learn subtle patterns specific to these factors that do not perfectly generalize to other datasets. Nevertheless, MetaCH’s performance on independent validation datasets remained within 10% of the cross-validation scores. Its strong performance on the Leal et al. dataset (auPR/auROC 0.89/0.84 compared to 0.8/0.73 for the Fairchild et al.^11^ model and 0.54/0.48 for the SSGAN^16^ model) is especially reassuring, as this data comes from gastric cancer patients—a cancer type not covered in the Razavi et al.^6^ matched cfDNA training dataset. Additionally, MetaCH’s consistent superior performance over existing ML models across all the external validation datasets highlights the advantage of strategies that leverage diverse sources of available information into a single learning framework.

The variation in performance might not only reflect limitations of the model but could also result from inconsistencies in ground truth labeling in datasets. Razavi et al.^6^, used to train the cfDNA-based classifier, had the highest depth of coverage among the cfDNA WBC-matched datasets. Lower depth in WBC sequencing could lead to missed variants, potentially misclassifying some CH variants as tumor derived. MetaCH demonstrated significantly poorer performance on the Zhang et al. dataset, which had a marked difference in sequencing depth between plasma (1000×) and WBC samples (400×), unlike other studies, which used higher and more consistent sequencing depths. This disparity likely contributed to misclassification, especially for low VAF variants, resulting in a high false-positive rate (also observed by Fairchild et al.^11^). While MetaCH might identify CH variants missed in the ground truth annotations of external datasets, the extent this effect is unclear. Further investigation using datasets with replicate matched controls and varying sequencing depths would help quantify this phenomenon.

Despite its promising results, MetaCH has limitations. Translating the model for clinical use requires establishing a relationship between reported metrics such as auROC and auPR with clinically meaningful outcomes. Ground truth experiments to correlate, for example, auPR with minimal residual disease false positive rates, are necessary for applying ML tools like MetaCH to the clinic. Future iterations of MetaCH could also incorporate patient-level features associated with CH, such as age^27^ and prior chemotherapy exposure^19^, as well as cfDNA-specific features such as fragmentomics^28^ or methylation^29^ to further improve the classification accuracy and clinical utility. Additionally, incorporating more panel-diverse annotated cfDNA samples in future training sets may improve the generalizability of this approach, as targeted sequencing, with its inherent genomic limitations, remains the primary approach in clinical cfDNA assays due to its high sensitivity required for translational research.

MetaCH represents a significant step towards more accurate CH variant identification in cfDNA samples. As we continue to refine and validate this approach, it has the potential to improve the accuracy of liquid biopsy-based cancer diagnostics and monitoring, ultimately contributing to more personalized and effective cancer care.

## Methods

### Datasets

This study utilizes multiple datasets for training and evaluating the classification of cfDNA variants (Fig. 1a). These datasets include publicly available blood and tumor sequencing data, as well as cfDNA samples collected from patients. The publicly available datasets were obtained from The Cancer Genome Atlas (TCGA), cBioPortal, and previously published studies. Each dataset varies in sequencing depth, variant composition, and patient cohort characteristics, providing a comprehensive foundation for model development and validation.

The details of each dataset are provided in the corresponding sections describing the framework components that utilize them.

### Mutational Enrichment Toolkit (METk): extracting general-purpose gene and variant embeddings

To generate embeddings for individual variants, genes, and cancer patients, we developed and applied the variants Enrichment Toolkit (METk), a framework that combines self-supervised natural language processing techniques with large-scale genomic datasets to create comprehensive, context-aware representations of genetic variants. METk is a Python API that takes in a table with pre-processed variants annotation format (MAF) files annotated with cancer type and returns variant embeddings (${E}_{v}$), gene embeddings (${E}_{g}$), patient-level variant embeddings (${E}_{{p}_{v}}$), patient-level gene embeddings (${E}_{{p}_{g}}$), and functional prediction scores of each variant (${E}_{f}$).

Mutational somatic signatures serve as a fingerprint for characterizing the underlying biology of cancer. Tumor somatic variants tend to leave a strong signature in the cancer cells that can indicate the multiple biological mechanisms present in the tumor (e.g., Aging, Tobacco smoking, APOBEC activity). In cfDNA samples, the enrichment of these signatures may indicate the origin of the variants^8,24,26^. As the prevalence of clonal hematopoietic variants is associated with the aging process, scoring the aging signature for individual variants can help to identify the origin of blood-derived variants. In the same context, identifying the tumor mutational signatures may help to identify the variants that are originated from the tumor. Therefore, identifying the mutational landscape from individual variants can contribute with the identification of its origin.

Prior research has demonstrated that larger variant sequence contexts can enrich information content of genomic data^30^, yet translating these contexts into a compact and meaningful set of numeric features remains an ongoing area of study. More recently, researchers have leveraged advances in natural language processing (NLP) to create a shared embedding space for patients and variants based on the larger-scale sequence context of variants^31^. Besides leveraging larger sequence context, studies have also explored generating gene embeddings based on co-occurrence patterns using NLP-inspired techniques^32,33^.

Inspired by these ideas, we developed the METk framework, which generates embeddings for genes and variants (Supplementary Fig. 2), in addition to scores that characterize variant impact on gene function, as described below.

### Variant embeddings

Variant embeddings are low-dimensional numerical representations (|*E*_*v*_*| = d*, i.e. a *d*−dimensional space) of genetic variants, capturing underlying patterns in variant data. These embeddings facilitate various downstream analyses, including variant classification, disease association studies, and variant effect prediction. To generate these embeddings, we leveraged the StarSpace^18^ model, a self-supervised learning framework originally developed for learning entity representations from structured data. This approach derives variant embeddings that encode biological context and co-occurrence patterns within patient variant profiles.

In this framework, each patient is represented as a set of genetic variants, analogous to an entity with multiple associated features. Therefore, the patient entity is modeled as a bag of all its variants, while the variant entity is described by a set of tokens, including the specific variant, its sequence context, associated gene, and cancer type (if available) (Fig. 1a). The model is trained using a contrastive learning approach, maximizing the similarity between co-occurring variants within the same patient while distinguishing them from randomly sampled negative variants.

To incorporate variant sequence context, we extract 2, 3, 4, and 5-mers from a 20-base pair window (10 bp upstream and 10 bp downstream from the variant’s location). During inference, cancer type is excluded as a feature to ensure the learned variant embeddings generalize across datasets.

For training, we utilized the pan-cancer TCGA dataset along with a subset of pan-cancer studies from cBioPortal (https://portal.gdc.cancer.gov, “msk_impact_2017”, “msk_ch_2020”, “tmb_mskcc_2018”, “pan_origimed_2020”, “pancan_pcawg_2020”, “msk_met_2021” downloaded from cBioPortal.org). This large-scale dataset enables the model to capture genomic, molecular, and clinical patterns, supporting applications such as variant effect prediction, cancer subtype classification, and patient stratification.

### Gene embeddings

Gene embeddings are low-dimensional vector representations that capture the functional relationships and co-variant patterns of genes. By mapping genes into a continuous embedding space, these representations encode biological relevance in a way that facilitates downstream tasks such as variant effect prediction, patient stratification, and pathway analysis. Inspired by the Continuous Bag-of-Words (CBOW) model from natural language processing (NLP), the Continuous Bag-of-Mutated Genes (CBMG) Model is a self-supervised approach for learning gene embeddings based on their co-occurrence in patient variant profiles. We used the pan-cancer mutational dataset from TCGA, where each entry is represented by a list of mutated genes, to train the model. In this framework, each patient is represented as a bag of mutated genes, analogous to a sentence composed of words. During training, a gene is randomly selected as the target, while the remaining mutated genes in the patient’s sample serve as the context. The model optimizes an objective function to predict the target gene based on its co-occurring genes, learning embeddings that capture underlying biological relationships, co-patterns, and functional similarity. The resulting gene embeddings can be utilized for various biomedical applications, including mutation-driven disease characterization and network-based gene function prediction.

### Patient-level gene and variant embeddings

For both gene and variant embeddings, patient-level embeddings are computed as the average of all gene or variant embeddings within each patient. Specifically, the patient-level variant embedding (${E}_{{p}_{v}}$) and patient-level gene embedding (${E}_{{p}_{g}}$) are obtained by averaging the embeddings of all variants or genes associated with a given patient, as illustrated in the figures for each embedding approach.

### Functional prediction scores

Functional prediction scores (${E}_{f}$) are extracted to provide additional information to the variant and gene embeddings. These scores are specifically obtained for non-synonymous variants, as they quantify the potential impact of variants on gene function and their likelihood of conferring a selective advantage in malignancies. We incorporated SnpEff^34^, a database that compiles prediction scores from multiple algorithms assessing the potential of amino acid substitutions on protein function, and SnpSift^35^, a tool that allows annotation and filtering of variants based on the SnpEff database. The resulting functional prediction scores (${E}_{f}$) offer an estimate of a variant’s potential contribution to cancer development.

### METk utilization

All these features are available through the Mutation Enrichment Toolkit (METk), a Python API designed to automatically extract the described embeddings. The METk framework supports the processing of both single nucleotide variants (SNVs) and insertions/deletions (INDELs), enabling a comprehensive analysis of mutation-derived embeddings. The final embedding dimensions used in MetaCH were $|{E}_{v}|=128$, ${{|E}}_{g}|=8$ and ${{|E}}_{f}|=37$.

### cfDNA-based classifier

The cfDNA-based classifier is a binary classifier which classifies each variant in a patient’s cfDNA sample as originating from CH or tumor (“cfDNA-Based Classifier,” Fig. 1b). This classifier was trained and validated using the cfDNA Razavi et al.^6^ dataset which includes annotations of variants as either tumor-derived or CH. These annotations were determined through matched plasma, tumor biopsy, and WBC sequencing from 124 patients with non-small cell lung, breast, or prostate cancer, where 53.2% of the patients harbored variants indicative of CH. Plasma and WBC were sequenced using a panel of 508 genes with >60,000x raw depth coverage. The study provides detailed annotation of cfDNA variants, including VAF, cancer type, and identifying their origin as blood or tumor. Variants of unknown significance that did not match either blood or tumor were excluded from our analysis. Specifically, 436 tumor somatic variants and 914 blood CH derived variants were used for training this classifier.

This classifier is optimized to distinguish between clonal hematopoiesis (CH) and tumor-derived variants in circulating cell-free DNA and provides a probability score (${S}_{{cfDNA}}$) which represents the likelihood of a given variant being classified as CH rather than tumor-derived. This score is subsequently integrated into the final stage of framework (the meta classifier), as shown in “Meta-Classifier,” Fig. 1a. Features used to train this classifier were variant and gene embeddings (${E}_{v}$, ${E}_{g}$), patient-level embeddings (${E}_{{Pv}}$, ${E}_{{p}_{g}}$), and functional prediction scores (${E}_{f}$) derived by METk, variant per patient-level (*VAF*), and cancer type (*Ct*).

### Sequence-based classifiers

Unlike the cfDNA-based classifier, the sequence-based classifiers were not trained on matched cfDNA data, but rather on data derived from blood and tumor samples. First, 77,068 tumor-derived variants were extracted from a study which sequenced tumors from 10,000 patients with 59 types of cancer (available at https://www.cbioportal.org/ “msk_impact_2017”). Second, 9810 blood-derived variants were extracted from a study which sequenced blood from 6650 patients (available at https://www.cbioportal.org/ “msk_ch_2020”). We annotated variants in our training data according to the CH subtype annotation described in Bolton et al.^19^. Their approach labeled variants based on their putative role in cancer pathogenesis using OncoKB^36^ and their recurrence in a dataset of myeloid neoplasms^37–39^. Specifically, 5800 variants were labeled as Clonal Hematopoietic putative cancer drivers (termed “CH-Oncogenic” in our study, equivalent to “CH-PD” for CH-putative driver in Bolton et al.). A total of 4010 blood variants were labeled as non-related to cancer pathogenesis (termed “CH-Non Oncogenic” in our study, an unnamed group in Bolton et al.).

After removing duplicate variants within each dataset independently, a total of 57,210 tumor variants, 2967 CH-Non Oncogenic, and 3778 CH-Oncogenic variants were used to train FlaML (Fast and Lightweight AutoML, a Python library that automates model selection and efficiently tunes hyperparameters^40^) to select and optimize two binary classifiers: The Sequence 1 classifier predicts CH-Oncogenic variants versus others (tumor or CH-Non-Oncogenic) and was trained using the CH-Oncogenic variants as the primary class while the CH-Non-Oncogenic and tumor variants were used as the secondary class. The Sequence 2 classifier predicts CH-Non-Oncogenic variants versus others (tumor or CH-Oncogenic) and was trained using the CH-Non-Oncogenic variants as the primary class while the CH-Oncogenic and tumor variants were used as the secondary class. The rationale for having two specialized binary classifiers within the space of these three categories is that CH-Oncogenic and CH-Non-Oncogenic variants exhibit distinct biological roles and decision boundaries. Training separate classifiers allows each model to optimize feature selection specific to its classification task, potentially improving prediction accuracy and interpretability.

Each binary classifier is optimized to learn features that distinguish the origins of CH subtypes, providing scores as probabilities of each variant belonging to either CH-Oncogenic (${S}_{{Sequence}1}$) or CH-Non-Oncogenic (${S}_{{Sequence}2}$).These scores are subsequently processed by the meta-classifier, as illustrated in “Meta-Classifier,” Fig. 1a.

The set of features used to train the sequence-based classifiers are the variant embeddings (${E}_{v}$), the gene level embeddings (${E}_{g}$), the functional prediction scores (${E}_{f}$) and the cancer type (*Ct*).

### Meta classifier

To integrate the complementary information from the cfDNA-based and sequence-based classifiers, we developed a metaclassifier that takes as input the probability scores from each of the three base classifiers for each variant (meta-features). The Meta-Classifier learns a function to distribute weights across various sources of evidence ${S}_{{Meta}}=f\left(\alpha {S}_{{cfDNA}}+\beta {S}_{{Sequence}1}+\gamma {S}_{{Sequence}2}\right)$. We used a logistic regression classifier to learn this function. To avoid overfitting, we used the same training and validation dataset splits employed in training the cfDNA-based classifier, as both classifiers were trained on the same data samples but with different features. The meta classifier predicts a final probability score (${S}_{{Meta}}$), which serves as the ultimate classification output, determining the likelihood of a variant being classified as CH.

### Model selection and performance evaluation

Each evidence-generating classifier was trained using FlaML (Fast and Lightweight AutoML), a Python library that automates model selection and efficiently tunes hyperparameters^40^. FlaML applies a range of classification algorithms (such as random forests, gradient boosting, and neural networks) to a task, systematically varying their hyperparameters and selecting the best-performing model based on cross-validated performance metrics.

We conducted stratified 5-fold cross-validation to evaluate the performance of each classifier in the training phase. Both the area under the Receiver Operating Characteristic curve (auROC) and the area under the Precision-Recall curve (auPR) were used and reported as evaluation metrics. Due to the class imbalance in most datasets, we primarily focused on auPR for result interpretation, while auROC is reported where relevant.

To provide a comprehensive evaluation on various external validation datasets, we have included both ROC and PR curves, which offer additional insights beyond their respective area-under-the-curve (AUC) values. ROC curves illustrate the trade-off between sensitivity and specificity, making them useful for understanding model performance across all classification thresholds. Meanwhile, PR curves highlight precision-recall dynamics, which are particularly relevant in settings with class imbalance, as they emphasize a model’s ability to identify positive instances without being misled by the abundance of negative samples. Together, these visualizations provide a detailed characterization of classifier performance, complementing the AUC metrics.

### Benchmarking datasets and methods

To assess the generalizability of MetaCH, we tested its performance on independent, publicly available cfDNA datasets representing a range of sequencing technologies, depths of coverage, post-sequencing analysis pipelines, cancer types, and clinical profiles^8,21–23^. All datasets included in our benchmarking studies utilized matched WBC sequencing such that all variants were annotated as either CH or tumor-derived by the studies^8,21–23^.

Chin et al.^22^ conducted ultradeep sequencing on cfDNA and WBCs from 100 breast cancer patients, equally divided between early-stage and advanced-stage disease. They used an amplicon-based panel with unique molecular tagging (UMT) to perform targeted-gene sequencing, covering 1021 cancer-related genes and achieving an average sequencing depth of approximately 4000x unique reads. Variant calling was performed using the Ion Reporter software. The study detected ctDNA in 43% of early-stage and 86% of advanced-stage patients, identifying 72 variants in 19 genes and finding a strong correlation of CH variants with age.

Chabon et al.^8^ analyzed 85 patients with stage I-III non-small cell lung cancer (NSCLC) using a personalized deep sequencing method. They matched tumor tissue, pretreatment plasma cfDNA, and leukocyte DNA for each patient, sequenced at a depth of 4000–5000×, and identified 909 variants from 255 genes. They used CAPP-seq with an error suppression model for variant calling and analysis. Similar to the Razavi et al. study, the majority of cfDNA variants in this cohort reflect CH and are non-recurrent. For our validation, we selected only the NSCLC variants labeled as absent (tumor) or present (blood) in matched leukocytes.

Leal et al.^23^ performed matched, targeted sequencing of cfDNA and matched WBCs of 50 gastric cancer patients (stages IB-IVA), both to a depth of 30,000×. They used VariantDx for variant calling and identified 224 variants in 28 genes.

Zhang et al.^21^ sequenced matched plasma and WBC samples from over 10,000 Chinese patients with various cancer types (stages I-IV) using parallel sequencing. They sequenced plasma at 1000× coverage and WBC at 400× coverage, using MutTect2 and quality filters for mutation calling and processing. The study used a targeted sequencing panel to detect ctDNA mutations. The mutation profiles varied across different cancer types, with certain mutations being more prevalent in specific cancers.

We compared the performance of MetaCH of these datasets with two existing machine learning approaches: Fairchild et al.^11^, which uses classical ML approaches on variant- and population-level features (including VAF, CH gene occurrence, and variant frequencies) in public databases and is trained on the Razavi et al. cfDNA with matched WBC dataset (similar to our cfDNA-based classifier), and SSGAN^16^, which uses a semi-supervised generative adversarial network that is sequence-based (similar to our sequence-based classifiers). Fairchild et al.^11^ provided the prediction scores and SSGAN^16^ made their implementation available. As both methods are designed specifically for predicting the origin of single nucleotide variants (SNVs), the external validation datasets were filtered accordingly.

## Supplementary information

Supplementary_Information

## Data Availability

Data from the pan-cancer TCGA dataset and all available studies from cBioPortal used to train METk are available at https://portal.gdc.cancer.gov and cBioPortal.org. The data used to train the sequence-based classifiers are available at https://www.cbioportal.org/ under “msk_impact_2017” and “msk_ch_2020” for the tumor and blood datasets, respectively. Data used to train the cfDNA classifer can be obtained directly from the Razavi et al.^6^. publication. External validation datasets can be obtained directly from the Chin et al.^22^., Chabon et al.^8^., Leal et al.^23^., and Zhang et al.^21^. publications. Alternative ML models used for comparison in our analyses can be obtained from the Fairchild et al.^11^. and SSGAN^16^ publications.

## Code availability

The MetaCH framework is available at https://github.com/gaarangoa/MetaCH. The METk package is available at https://github.com/gaarangoa/METk.
