# Supplementary material for: An artificial intelligence-based model for prediction of clonal hematopoiesis variants in cell-free DNA samples
Source: NPJ Precis Oncol. 2025 May 20;9:147. doi: 10.1038/s41698-025-00921-w (PMC12092662; doi:10.1038/s41698-025-00921-w)
Supplement: Supplementary file 1 — Supplementary_Information [file 41698_2025_921_MOESM1_ESM.pdf]

Supplementary Table 1: K-fold (k=5) Cross-validation performance of each framework's component/classifier

Classifier	Stage	Training Dataset	Annotations/ Classes (N)	auROC (mean$\pm$std)	auPR (mean$\pm$std)
**cfDNA**	2	Razavi et al.	CH vs. Tumor (914, 436)	0.93 ± 0.01	0.96 ± 0.01
**Sequence 1**	2	MSK	CH-Oncogenic vs. others (2967, 60988)	0.96 ± 0.003	0.78 ± 0.01
**Sequence 2**	2	MSK	CH-Non-Oncogenic vs. others (3778, 60177)	0.80 ± 0.01	0.27 ± 0.01
**Meta-Classifier**	3	Razavi et al.	CH vs. Tumor (914, 436)	0.93 ± 0.01	0.97 ± 0.01

Supplementary Table 2: Performance of the MetaCH framework and comparison with the baseline methods on each independent validation dataset

Dataset	Annotations (N)		MetaCH	Fairchild et al.	SSGAN
**Zhang et al.**	CH vs Tumor (262, 3391)	**auROC**	0.64	0.63	0.50
		**auPR**	0.20	0.13	0.07
		**Precision**	0.12	0.12	0.05
		**Recall**	0.51	0.58	0.15
		**F1**	0.19	0.20	0.08
**Chin et al.**	CH vs Tumor (17, 57)	**auROC**	0.84	0.69	0.56
		**auPR**	0.69	0.47	0.34
		**Precision**	0.29	0.30	0.28
		**Recall**	1	1.00	0.41
		**F1**	0.45	0.46	0.33
**Chabon et al.**	CH vs Tumor (325, 235)	**auROC**	0.84	0.83	0.53
		**auPR**	0.88	0.84	0.58
		**Precision**	0.71	0.73	0.51
		**Recall**	0.92	0.91	0.09
		**F1**	0.80	0.81	0.16
**Leal et al.**	CH vs Tumor (108, 84)	**auROC**	0.82	0.73	0.71
		**auPR**	0.88	0.80	0.71
		**Precision**	0.62	0.61	0.70
		**Recall**	0.96	0.91	0.30

		**F1**	0.75	0.73	0.42

Supplementary Table 3: Description of features inputted to the classifiers

Feature	Derived by	Inputted to	Embedding size
**Variant embedding ($E\mathrm{v}$)**	MetK	Sequence 1, 2, cfDNA classifiers	128
**Gene embedding (Eg)**	MetK	Sequence 1, 2, cfDNA classifiers	8
**Patient-level variant embedding ($E\mathrm{p_v}$)**	MetK (average of variant embeddings for all variants within each patient)	cfDNA classifier	128
**Patient-level gene embedding ($E\mathrm{p_g}$)**	MetK (average of gene embeddings for all mutated genes within each patient)	cfDNA classifier	8
**Functional prediction scores (Ef)**	MetK	Sequence 1, 2, cfDNA classifiers	37
**Variant per patient-level VAF**	Dataset info (Razavi et al.)	cfDNA classifier	1
**Cancer type**	Dataset info (Razavi et al., Bolton et al., Zehir et al.)	Sequence 1, 2, cfDNA classifiers	1

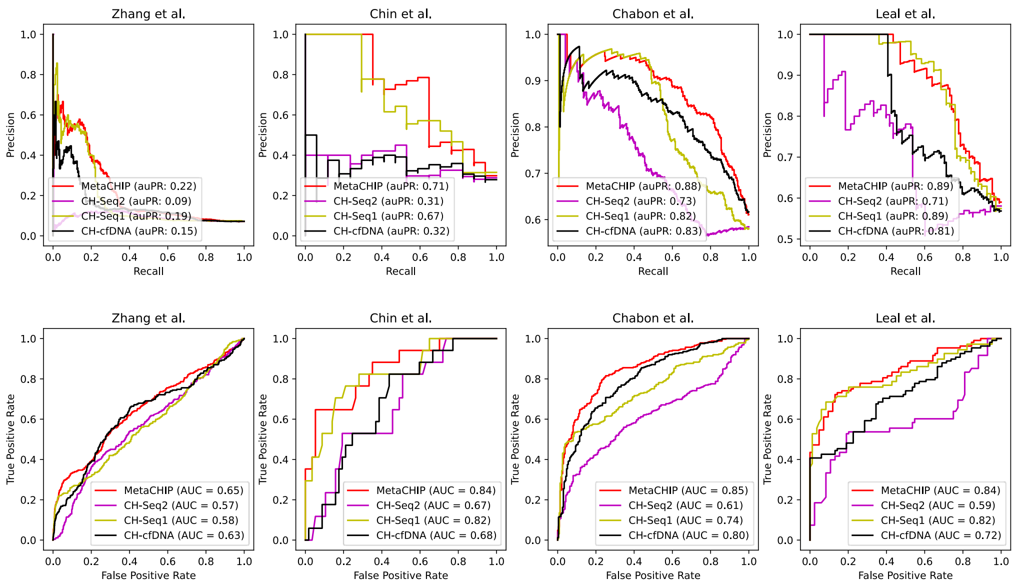

Supplementary Figure 1. Precision-recall curves for MetaCH and individual classifiers across external cfDNA validation datasets. MetaCH outperforms average of the individual classifiers (CH-Seq1, CH-Seq2, and CH-cfDNA) in predictive power for determining variant origin in four independent validation datasets. The area under the precision-recall curve (denoted by auPR) is indicated for each classifier, highlighting MetaCH's consistently higher performance.

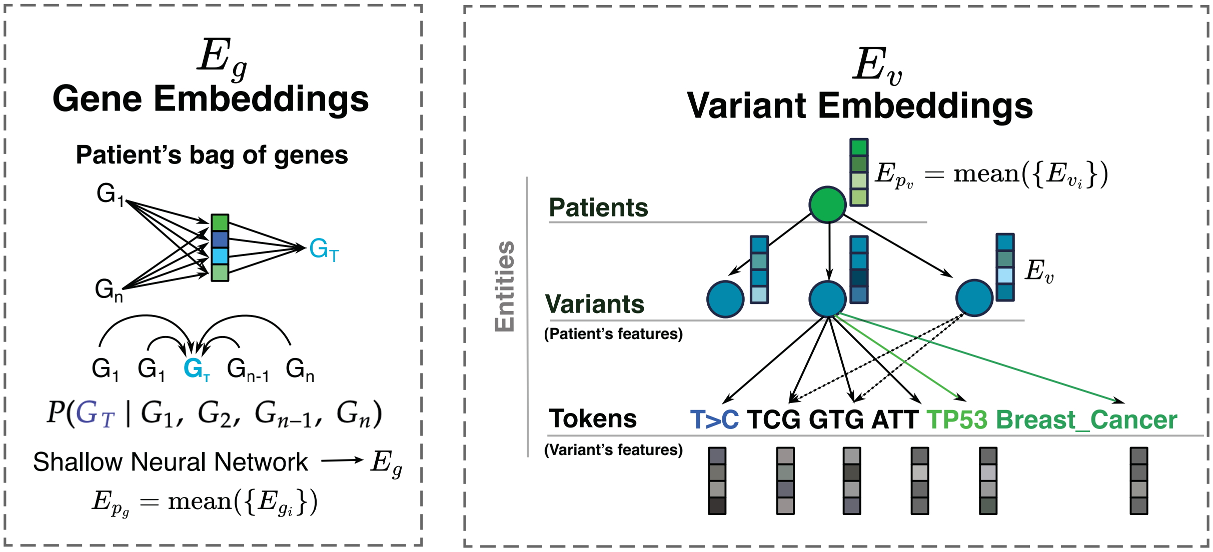

Supplementary Figure 2. Schematic of gene and variant embeddings extracted by the Mutational Enrichment Toolkit (METk).
